# Gut microbiota and protein-to-protein ratios in NAFLD: insights from Mendelian randomization and murine studies

**DOI:** 10.3389/fnut.2025.1597390

**Published:** 2025-07-18

**Authors:** Langhuan Lei, Wei Shi, Xing Yang, Jiali Lin, Qiuyu Liang, Xiaozhi Huang, Liuxian Pan, Wei Li

**Affiliations:** ^1^Research Center of Health Management, Guangxi Zhuang Autonomous Region People's Hospital and Guangxi Academy of Medical Sciences, Nanning, China; ^2^Department of Health Management, Guangxi Zhuang Autonomous Region People's Hospital and Guangxi Academy of Medical Sciences, Nanning, China

**Keywords:** gut microbiota, protein-to-protein ratios, NAFLD, Mendelian randomization, *Lactobacillus salivarius*

## Abstract

**Background:**

Gut microbiota and protein metabolism play critical roles in non-alcoholic fatty liver disease (NAFLD) progression, but their causal relationships remain unclear. This study integrates Mendelian randomization (MR) analysis and experimental validation to identify microbial and molecular contributors to NAFLD and explore potential therapeutic targets.

**Methods:**

Two-sample MR analysis was performed to assess the causal effects of gut microbiota and protein-to-protein ratios on NAFLD using inverse variance-weighted, maximum likelihood, MR-Egger, weighted median, weighted mode, and Wald ratio methods. Sensitivity analyses were conducted to ensure result robustness. Mediation analysis was applied to examine whether protein-to-protein ratios mediate the link between gut microbiota and NAFLD.

**Results:**

MR analysis identified 19 gut microbial taxa and 148 protein-to-protein ratios significantly associated with NAFLD. Additionally, 49 significant mediation relationships were identified, where seven gut microbial taxa influenced NAFLD via 45 protein-to-protein ratios. MR analysis identified 38 proteins significantly associated with NAFLD, derived from 192 unique proteins involved in 148 NAFLD-related protein-to-protein ratios. Experimental validation confirmed the protective role of *Lactobacillus salivarius*, which alleviated hepatic lipid accumulation, improved glucose-lipid metabolism, and reduced inflammatory cytokine expression. Among the identified targets, the hepatic mRNA expression levels of ANGPT1, SKAP2, SPARC, and STAMBP were significantly upregulated in NAFLD tissues and were markedly reduced following *Lactobacillus salivarius* supplementation.

**Conclusion:**

This study establishes a causal link between gut microbiota, protein metabolism, and NAFLD, identifying microbial and molecular targets for intervention. The findings support microbiota-based therapies and protein biomarkers for NAFLD management, warranting further clinical validation.

## 1 Introduction

Non-alcoholic fatty liver disease (NAFLD) affects ~25% of adults worldwide, progressing from simple steatosis to non-alcoholic steatohepatitis (NASH), fibrosis, and hepatocellular carcinoma (HCC) (1). While its pathogenesis is multifactorial, gut microbiota has emerged as a critical regulator of hepatic lipid metabolism, inflammation, and insulin sensitivity. Specific microbial taxa exhibit distinct roles in disease progression (2). *Clostridium* species have been implicated in gut barrier dysfunction, increased lipopolysaccharide (LPS) translocation, and hepatic inflammation, all of which contribute to NAFLD pathophysiology (3). In contrast, *Bacteroides* species play a pivotal role in bile acid metabolism and lipid absorption, with certain strains potentially exerting protective effects against hepatic steatosis (4). Microbial-derived proteins, including bacterial extracellular vesicle proteins, lipoproteins, and adhesins, influence hepatic homeostasis by modulating farnesoid X receptor (FXR), peroxisome proliferator-activated receptor (PPAR), and Toll-like receptor (TLR) signaling, thereby regulating lipid metabolism, insulin sensitivity, and immune responses (5). Despite these associations, the precise microbial contributors and mechanisms driving NAFLD remain insufficiently understood, necessitating further investigation to clarify their roles in disease pathogenesis and potential therapeutic applications.

Protein-to-protein ratios (PPRs) serve as dynamic metabolic biomarkers, reflecting systemic adaptations beyond single-protein measurements. Within the gut-liver axis, microbiota-modulated proteins influence hepatic lipid metabolism, inflammation, fibrosis, and insulin signaling, all of which are central to NAFLD pathogenesis (6, 7). Numerous studies have linked plasma protein alterations to NAFLD severity, with dysregulated lipoproteins, apolipoproteins, cytokines, and extracellular matrix proteins contributing to disease progression (8). For instance, disturbances in Apolipoprotein B/A1 ratios have been associated with hepatic lipid accumulation, while fibrinogen and complement proteins are implicated in NAFLD-related inflammation and fibrosis (9). Moreover, insulin resistance-related proteins, such as IGFBP and fetuin-A, modulate glucose metabolism and hepatic steatosis (10). Given that gut microbiota profoundly influence host protein metabolism through bile acid signaling, immune modulation, and metabolic reprogramming, proteins may serve as both biomarkers and mediators of disease progression (11, 12). However, the causal relationships between PPRs and NAFLD remain poorly defined.

Mendelian randomization (MR) leverages genetic variants as instrumental variables (13), offering a robust framework to infer causality in gut microbiota-NAFLD interactions while minimizing confounding (14). Mediation analysis is a statistical approach used to uncover causal pathways by identifying intermediate variables that link an exposure to an outcome (15). In the context of NAFLD, gut microbiota may exert their effects indirectly through PPRs intermediaries, which reflect complex regulatory interactions in hepatic metabolism. Murine models provide an experimental platform to validate these causal pathways, enabling precise modulation of microbial composition via probiotic intervention.

**Figure 1:**
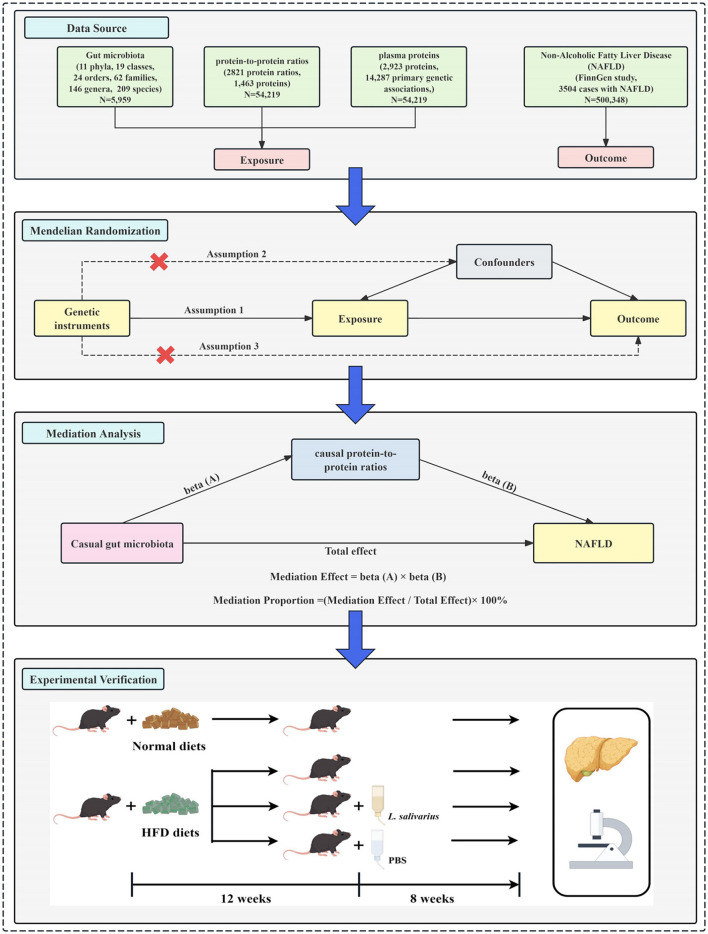
Flowchart of the study. (1) Data sources. Summary statistics for gut microbiota, protein-to-protein ratios, plasma protein levels, and non-alcoholic fatty liver disease (NAFLD) were obtained from publicly available genome-wide association studies (GWAS). (2) Mendelian randomization (MR) analysis. A two-sample MR framework was implemented to assess the causal relationships between gut microbiota, protein ratios, plasma proteins, and NAFLD. The MR analysis was based on three core assumptions (1): genetic instruments are associated with the exposure; (2): genetic instruments are not associated with confounders; (3): genetic instruments influence the outcome exclusively through the exposure. (3) Mediation analysis. Mediation analysis was conducted to explore whether protein-to-protein ratios act as mediators between gut microbiota and NAFLD. The causal pathways were quantified using two regression coefficients: beta (A) for the effect of gut microbiota on protein ratios, and beta (B) for the effect of protein ratios on NAFLD. The total effect was partitioned into direct and mediated effects. (4) Experimental validation. A high-fat diet (HFD)-induced NAFLD mouse model was established. After 12 weeks of HFD feeding, mice were orally administered *Lactobacillus salivarius* or phosphate-buffered saline (PBS) for 8 weeks. Liver tissues were collected for histological examination and qPCR analysis to evaluate gene expression changes following *Lactobacillus salivarius* supplementation in HFD-induced NAFLD mice.

While the gut-liver axis is increasingly recognized in NAFLD, the mechanisms linking gut microbiota, protein metabolism, and disease progression remain unclear. PPRs, based on circulating plasma proteins, have been linked to NAFLD severity and progression. However, their biological significance and therapeutic potential warrant further investigation. This study integrates MR, mediation analysis, and murine validation to uncover causal links between gut microbiota, protein metabolism, and hepatic dysfunction. The study design is depicted in Figure 1. These findings may advance biomarker discovery and microbiota-targeted therapies, offering new insights into NAFLD pathogenesis.

## 2 Materials and methods

### 2.1 Study design

The study was conducted in three phases (Figure 1). (1) GWAS summary statistics were obtained from publicly available datasets, including gut microbiota, protein-to-protein ratios, plasma proteins, and NAFLD. (2) Two-sample MR analyses were performed to assess the causal relationships among gut microbiota, protein-to-protein ratios, plasma proteins, and NAFLD. Additionally, a two-step MR approach was used to evaluate whether protein-to-protein ratios mediate the causal effect of gut microbiota on NAFLD. (3) Mediation Analysis: Mediation analysis was conducted to quantify the indirect effect of gut microbiota on NAFLD through causal PPRs, using the product of coefficients method. (4) Murine experiments were performed to evaluate the effects of *L. salivarius* on NAFLD progression, followed by histological and qPCR analyses of liver tissues.

### 2.2 Data sources

Summary statistics from GWAS on gut microbiota were sourced from the largest multi-ethnic meta-analysis available in the GWAS Catalog (GCST90032172 to GCST90032644). This dataset includes 2,801 microbial taxa and 7,967,866 genetic variants from 5,959 individuals in the FR02 cohort. A total of 471 summary statistics were used, spanning 11 phyla, 19 classes, 24 orders, 62 families, 146 genera, and 209 species (16). The genetic data on plasma protein-to-protein ratios are derived from the latest GWAS study, which provides new insights into 2,821 protein ratios and their associations with diseases (17). Genetic association information for circulating plasma proteins (pQTLs) was sourced from the UK Biobank Protein Project (UKB PPP) (18). Genetic association data for NAFLD were obtained from the FinnGen cohort (finngen-R12-NAFLD), which includes 3,504 patients and 496,844 controls. NAFLD cases were defined as individuals with at least one record of ICD-10 code K76.0 [Fatty (change of) liver, not elsewhere classified]. Individuals with diagnoses of viral hepatitis (B15–B19), alcoholic liver disease (K70), autoimmune liver diseases (K75.4, K74.3), liver cirrhosis (K74.6), or liver cancer (C22) were excluded. Controls were selected as individuals without any diagnosis of liver-related diseases. All phenotypic information was derived from nationwide hospital discharge and outpatient records based on electronic health records (19).

To identify differentially expressed genes, expression data were obtained from the GepLiver database (http://www.gepliver.org/) for the comparison between normal and NAFLD liver tissues, and from the GSE164760 dataset for the comparison between non-malignant NASH liver tissues and tumor tissues from patients with NASH-associated hepatocellular carcinoma (NASH-HCC).

### 2.3 Selection of instrumental variables (IVs)

In conventional two-sample MR analysis, the selection criteria for instrumental variables are as follows (20, 21): (1) SNPs with a *p*-value <5 × 10^−^8 are chosen, with the linkage disequilibrium coefficient (*r*^2^) set to 0.001 and a linkage disequilibrium region of 10,000 kb. If genome-wide significant loci are insufficient, SNPs with *p* < 1 × 10^−^5 are used as alternatives (22). The minor allele frequency (MAF) must be >0.01 to ensure the independence of each SNP and eliminate the influence of linkage disequilibrium; (2) the instrumental variables must not exhibit pleiotropy; (3) the instrumental variables must be independent of potential confounders; (4) preference is given to instrumental variables with an *F*-statistic >10 to minimize bias from weak instruments; (5) Steiger filtering removed instrumental variables (IVs) more strongly correlated with the outcome than the exposure, ensuring accurate mediation.

### 2.4 Mendelian randomization analysis

#### 2.4.1 Two-sample Mendelian randomization

We employed MR to evaluate causal relationships between gut microbiota, the protein-to-protein ratio, protein abundance, and NAFLD. For single instrumental variables (IVs), the Wald ratio estimator was used, while methods like Inverse-Variance Weighted (IVW) (23), maximum likelihood, MR-Egger (24), weighted median (25), and weighted mode (26) were applied for multiple IVs. IVW, our primary method, combines Wald ratio estimates through meta-analysis. Sensitivity analyses, including MR-Egger and MR-PRESSO, were conducted to check for pleiotropy. Heterogeneity was evaluated using Cochran's *Q*-test (27), and the Steiger filter excluded IVs more strongly associated with the outcome than the exposure. The Benjamini–Hochberg procedure corrected the false discovery rate (FDR) for multiple comparisons. All analyses were performed in R (version 4.4.3).

#### 2.4.2 Mediation analysis

Mediation analysis was conducted to investigate whether protein-to-protein ratios mediate the causal relationship between gut microbiota and NAFLD. First, the causal effects of gut microbiota on protein-to-protein ratios were estimated using two-sample MR to obtain beta (A). Next, the causal effects of protein-to-protein ratios on NAFLD were assessed using two-sample MR to obtain beta (B). The mediation effect was calculated as beta (A) × beta (B), while the total effect of gut microbiota on NAFLD was derived from the primary MR analysis. The direct effect was estimated as total effect–mediation effect, and the mediation proportion was determined using the formula: mediation proportion = (mediation effect/total effect) × 100%. The 95% confidence intervals (CIs) for the mediation effects and proportions mediated were calculated using the delta method.

### 2.5 Experimental validation in mice

Female C57BL/6 mice (6–8 weeks old, specific pathogen-free) were housed under controlled temperature and humidity with *ad libitum* access to food and water. After a 7-day acclimatization period, the mice were randomly assigned to four groups (*n* = 10 per group): normal diet (ND), high-fat diet (HFD), HFD plus *Lactobacillus salivarius* (*L. salivarius*), and HFD plus PBS (PBS). The high-fat diet contained 40% fat. *Lactobacillus salivarius* Li01 was used as the probiotic intervention strain. Mice in the treatment groups received *Lactobacillus salivarius* or PBS (10^9^ CFU/ml, 0.2 ml per mouse) via intragastric gavage three times per week for eight consecutive weeks, concurrent with a 20-week high-fat diet regimen. Detailed protocols for bacterial culture, serological assays, histological staining, western blotting, RNA extraction and quantitative real-time PCR (qRT-PCR) are provided in Supplementary Material 2.

All animal experiments were approved by the Ethics Committee of Guangxi Zhuang Autonomous Region People's Hospital (Approval Number: KY-GZR-2024-093) and conducted in accordance with institutional guidelines for animal care and use.

### 2.6 Statistical analysis

Statistical analyses were performed using GraphPad Prism version 10.0, and results were expressed as the mean ± standard error of the mean (SEM). Student's *t*-test was conducted for data with normal distribution and homogeneous variance; otherwise, Welch's *t*-test was employed. Multiple-group comparisons were performed using one-way ANOVA, followed by *post-hoc* tests, with statistical significance defined as *p* < 0.05. Data not meeting assumptions of normality were analyzed using the Kruskal–Wallis test.

## 3 Results

### 3.1 Causal relationships between gut microbiota and NAFLD

**Figure 2:**
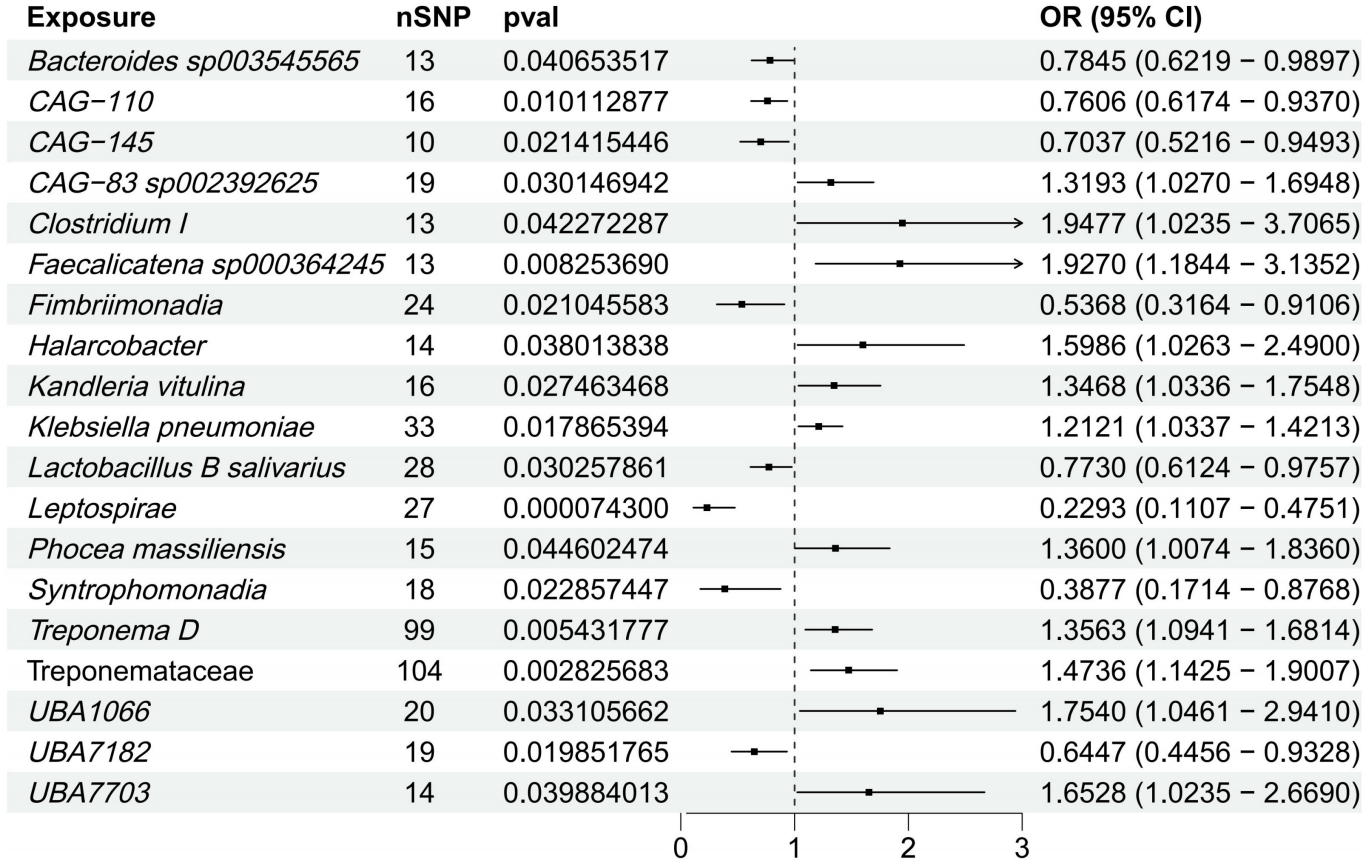
Forest plot of the causal relationship between gut microbiota and NAFLD. This forest plot depicts the causal association between gut microbiota and NAFLD. The horizontal bars indicate the odds ratio (OR) and 95% confidence interval (CI) for the impact of gut microbiota on NAFLD, as estimated by the IVW method.

Through two-sample MR, we identified 19 significant associations between gut microbiota and NAFLD (Figure 2, Supplementary Table S1). The *Lactobacillus B salivarius* and *Bacteroides sp003545565* exhibited an inverse association with NAFLD. Notably, *Clostridium I and Klebsiella pneumoniae* demonstrated a positive correlation with NAFLD. After FDR correction, only class *Leptospirae* showed a significant negative correlation with NAFLD (FDR = 0.0351). Sensitivity analyses corroborated these findings (Supplementary Table S2), and reverse MR revealed no significant association between the gut microbiome and NAFLD (Supplementary Tables S3, S4).

### 3.2 Causal relationships between protein-to-protein ratios and NAFLD

**Figure 3:**
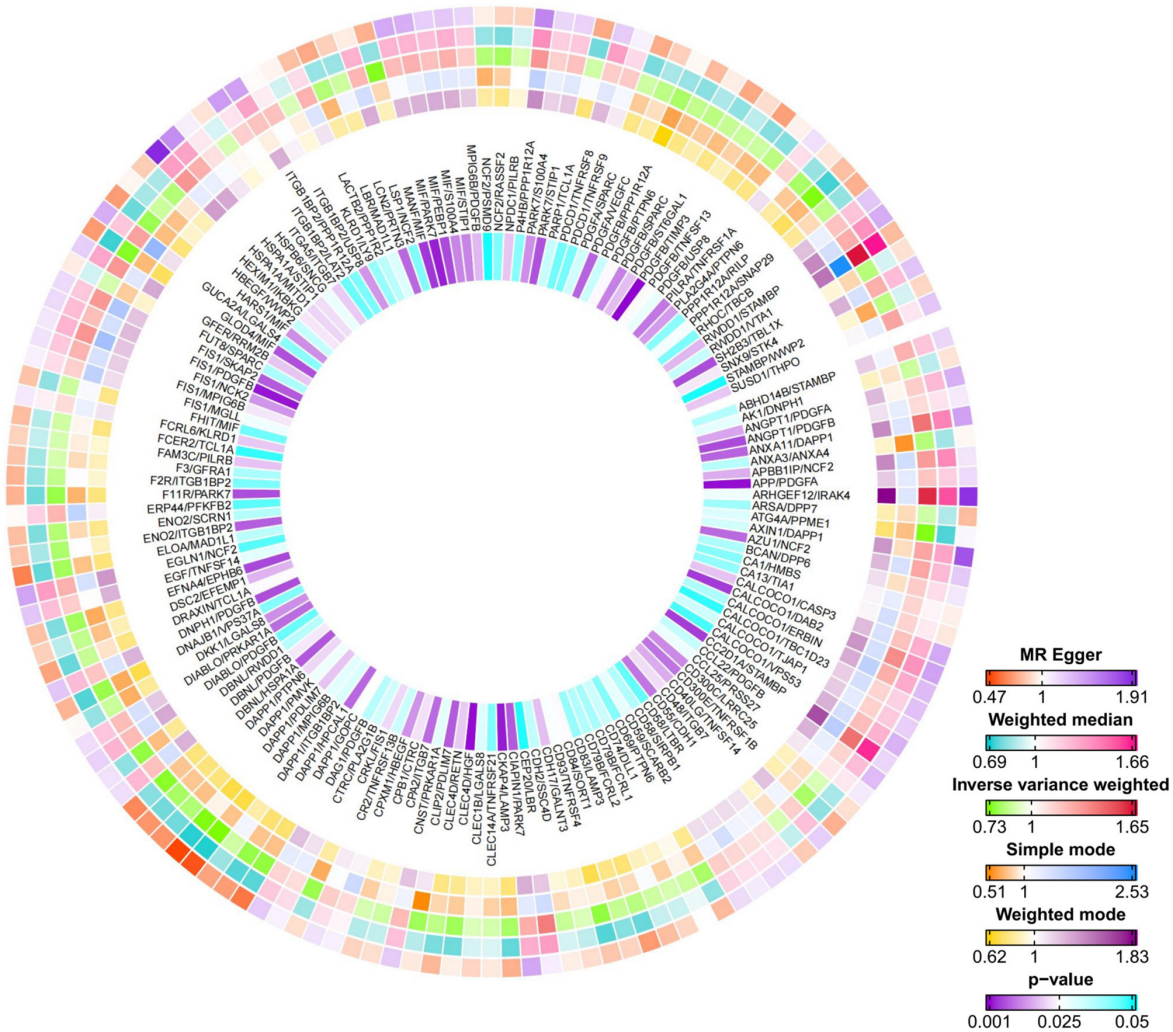
Circos heatmap of the Mendelian randomization analysis between protein ratio and NAFLD. From the outermost to the innermost ring, the heatmap represents effect estimates derived from the MR Egger, Weighted Median, Inverse Variance Weighted (IVW), Simple Mode, and Weighted Mode methods, respectively. The innermost ring displays the corresponding *p*-values. The color gradients reflect effect sizes, with white indicating a null effect (OR = 1).

After performing inverse variance weighting (IVW) analysis on 2,821 plasma protein-to-protein ratios, we identified 148 ratios with a causal relationship to NAFLD (Figure 3, Supplementary Table S5). Among these findings, the PDGFB, DAPP1 and MIF protein were identified as being associated with several other proteins, thereby influencing NAFLD. The results of heterogeneity and horizontal pleiotropy analyses are summarized in Supplementary Table S6. The protein-to-protein ratios provide a novel approach to studying the pathogenesis of metabolic liver diseases, including NAFLD.

### 3.3 Mediation analysis results

Mediation analysis was conducted to investigate the causal pathways linking gut microbiota to protein-to-protein ratios in NAFLD. Using two-sample MR, we identified 49 significant associations between gut microbiota and protein-to-protein ratios in NAFLD (Supplementary Table S7). Sensitivity analyses confirmed the robustness of the MR results, with no evidence of heterogeneity or pleiotropy (Supplementary Table S8). The analysis identified a significant mediation effect of EFNA4/EPHB6 in the relationship between *Phocea massiliensis* and NAFLD (β = 0.0764, 95% CI [0.00871, 0.144], *p* = 0.027), accounting for 24.84% of the total effect.

### 3.4 Causal association between plasma proteins and NAFLD

**Figure 4:**
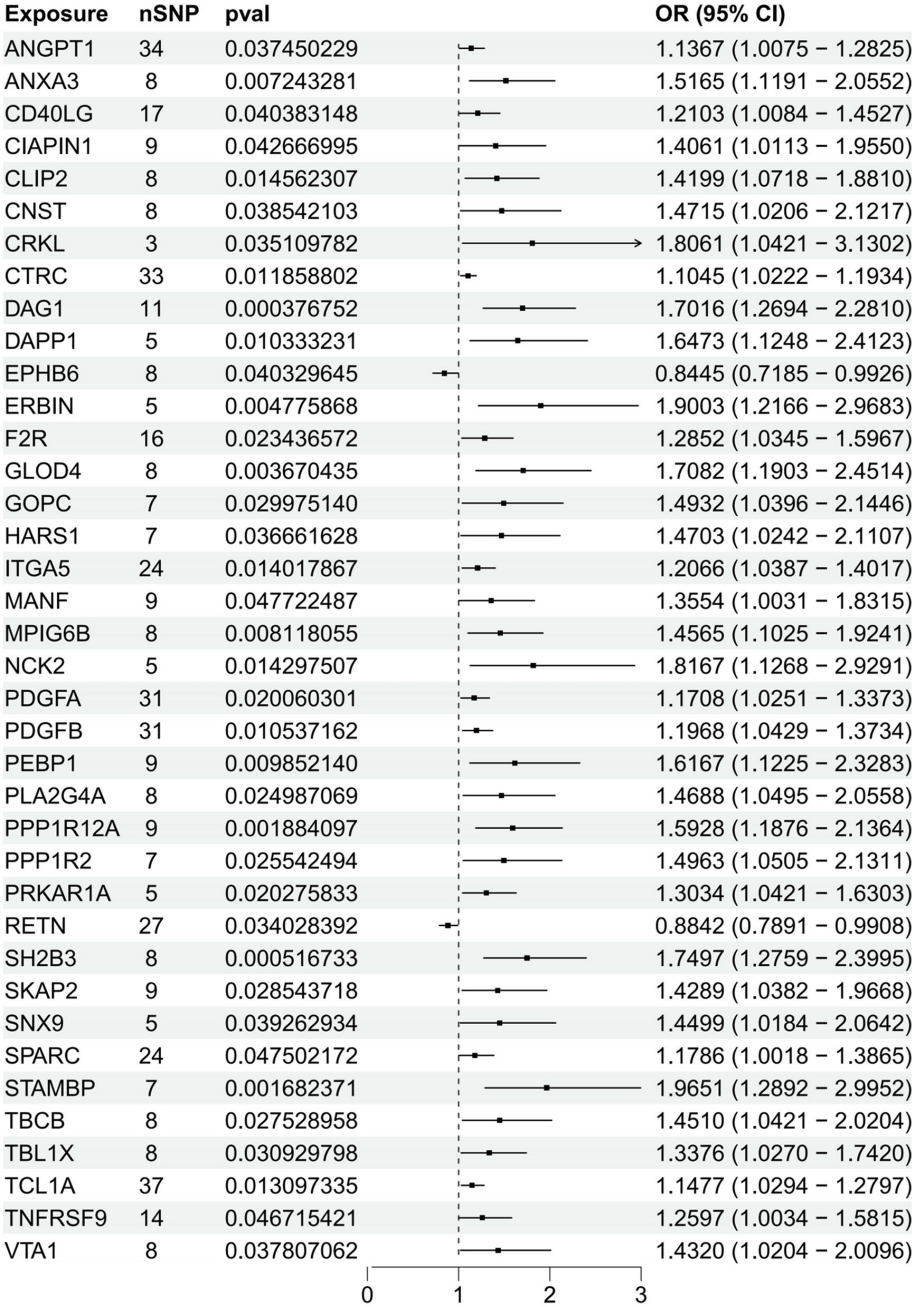
Forest plot of the causal relationship between plasma protein levels and NAFLD. This forest plot presents the estimated causal effects of plasma protein levels on NAFLD. The horizontal bars depict the odds ratio (OR) and 95% confidence interval (CI) for plasma protein' impact on NAFLD, as determined by the IVW approach.

MR analysis was conducted to assess causal associations between NAFLD and plasma proteins derived from 148 NAFLD-associated protein-to-protein ratios (PPRs). After removing duplicates, a total of 192 unique proteins involved in these PPRs were identified. Each of these 192 proteins was subsequently subjected to individual two-sample MR analysis to evaluate its direct causal relationship with NAFLD. Based on a significance threshold of *p* < 0.05, 38 proteins were identified as significantly associated with NAFLD. Among these, 36 proteins were found to promote the development of NAFLD (e.g., ANGPT1, ANXA3, CD40LG, CRKL, F2R, GOPC, ITGA5, PDGFA, PDGFB, SKAP2, SPARC, and TNFRSF9), while two proteins, RETN and EPHB6, exhibited protective effects by mitigating disease progression (Figure 4, Supplementary Table S9). Sensitivity and heterogeneity analyses of the protein quantitative trait loci (pQTLs) associated with NAFLD are provided in Supplementary Table S10.

### 3.5 *Lactobacillus salivarius* alleviate glycolipid metabolism, improves hepatic function, and reduces inflammation

**Figure 5:**
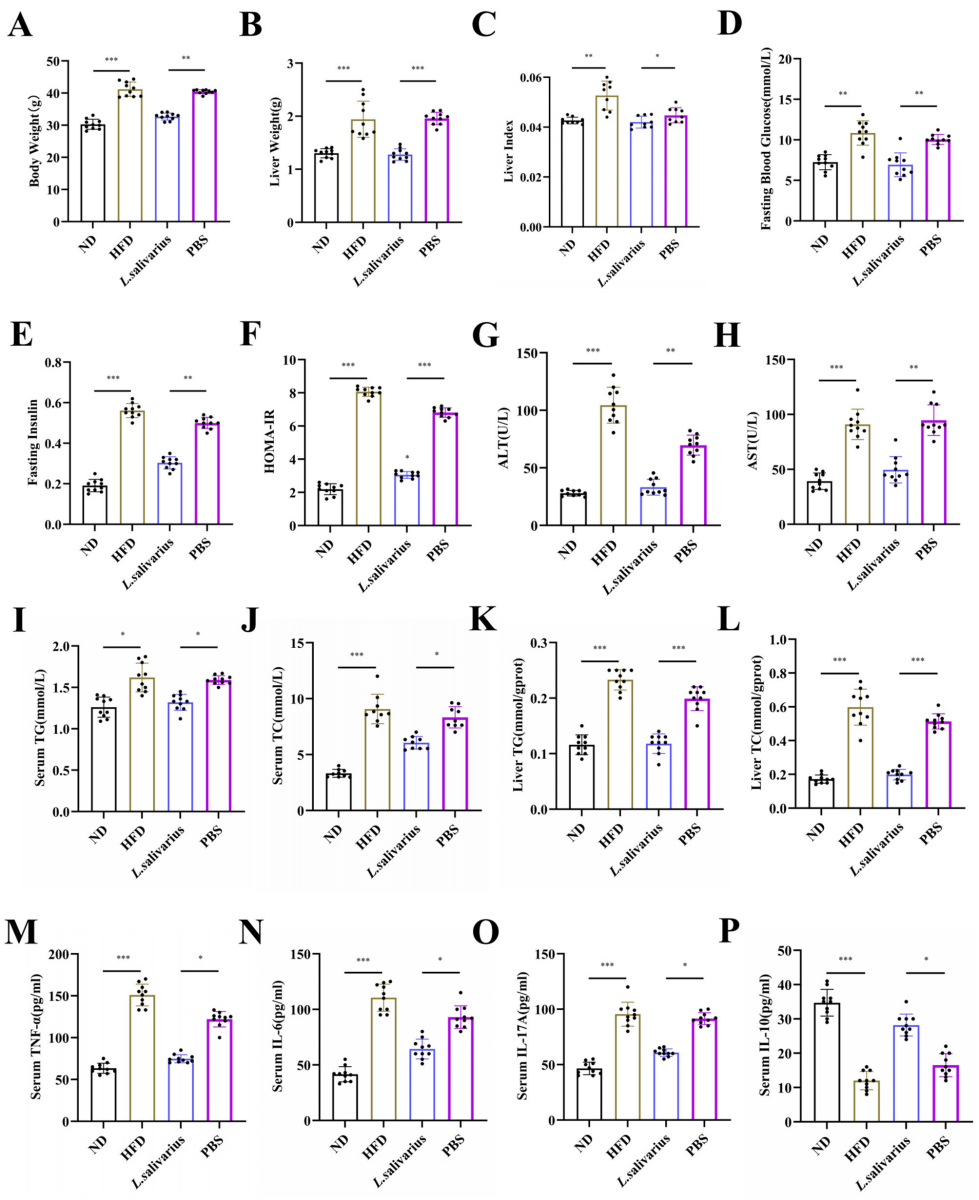
Effects of *Lactobacillus salivarius* on glycolipid metabolism indicators. **(A)** Final body weight of mice prior to sacrifice, after 20 weeks on a high-fat diet (HFD). **(B)** liver mass and **(C)** liver index (liver weight/body weight). Fasting blood glucose **(D)** and insulin concentrations **(E)**. **(F)** Calculation of HOMA-IR (homeostasis model assessment for insulin resistance; HOMA-IR = fasting blood glucose × fasting insulin/22.5). **(G)** Serum concentrations of alanine aminotransferase (ALT), **(H)** aspartate aminotransferase (AST), **(I)** triglycerides (TG), and **(J)** total cholesterol (TC). Liver tissue TG **(K)** and TC **(L)** concentrations. Serum concentrations of TNF-α **(M)**, IL-6 **(N)**, IL-17A **(O)**, and IL-10 **(P)**. *L. salivarius* refers to *Lactobacillus salivarius*. Data are expressed as the mean ± standard error of the mean (SEM), with *N* = 10 per group. Statistical significance is denoted as ^*^p < 0.05, ^**^p < 0.01, and ^***^p < 0.001.

After a 7-day acclimation period, no significant differences in body weight were observed among the groups (Supplementary Figure S1A). Compared to the ND group, mice fed with HFD or PBS developed pronounced metabolic dysfunction, including increased body weight, liver weight, liver index, and fasting blood glucose (Figures 5A–D), along with elevated fasting insulin, HOMA-IR, and serum ALT and AST levels, indicating insulin resistance and liver injury (Figures 5E–H). *Lactobacillus salivarius* treatment markedly ameliorated these abnormalities. Moreover, *L. salivarius* significantly reduced serum and hepatic triglyceride (TG) and total cholesterol (TC) levels, thereby alleviating hepatic steatosis (Figures 5I–L). Inflammatory cytokine profiling revealed increased TNF-α, IL-6, and IL-17A and decreased IL-10 in HFD and PBS groups, consistent with NAFLD-associated inflammation. These changes were reversed by *L. salivarius*, which suppressed pro-inflammatory cytokines and restored IL-10 levels (Figures 5M–P), highlighting its anti-inflammatory potential.

### 3.6 *Lactobacillus salivarius* alleviates HFD-Induced hepatic steatosis

**Figure 6:**
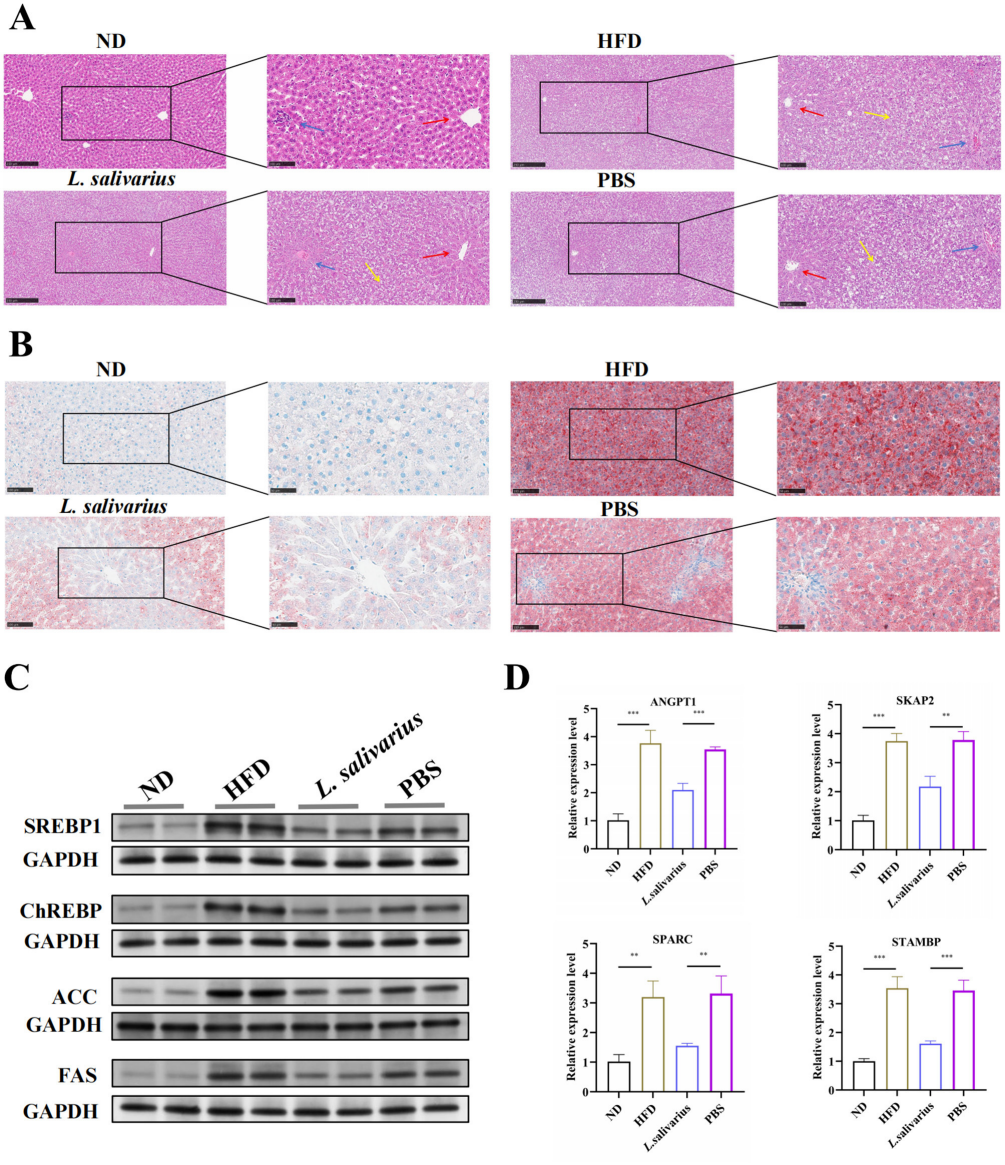
*Lactobacillus salivarius* mitigates liver lipid metabolism disruptions. **(A)** Representative micrographs of mouse liver sections stained with hematoxylin and eosin (H&E). Central vein (red arrows), portal triad (blue arrows), and hepatic steatosis (yellow arrows) are indicated. Images were captured at 10 × and 20 × (magnification). **(B)** Micrographs of mouse liver sections stained with Oil Red O, at 20 × and 40 × magnification. **(C)** Western blot analysis of SREBP-1, ChREBP, ACC, and FAS proteins. **(D)** Quantitative real-time PCR (qPCR) analysis of hepatic mRNA expression levels of ANGPT1, SKAP2, SPARC, and STAMBP in liver tissues from the experimental groups. Data are presented as mean ± SEM (*N* = 3 per group). Statistical significance: ^**^p < 0.01, and ^***^p < 0.001.

Histological examination (HE staining) showed normal liver architecture in the ND group, while evident disruption, hepatocellular swelling, and lipid accumulation, typical of NAFLD, were observed in the HFD and PBS groups. Treatment with *L. salivarius* notably reduced hepatocellular swelling and lipid accumulation, indicating significant alleviation of hepatic steatosis (Figure 6A, Supplementary Figures S1B, D, E). These observations were further confirmed by Oil Red O staining, demonstrating decreased lipid deposition in the *L. salivarius*-treated mice (Figure 6B, Supplementary Figure S1C). Protein analyses showed elevated hepatic expression of lipid synthesis-related proteins, including SREBP1, ChREBP, ACC, and FAS in the HFD and PBS group; these levels were significantly reduced following *L. salivarius* administration, indicating reduced hepatic lipid synthesis and accumulation (Figure 6C).

### 3.7 Validation of candidate gene expression of NAFLD

To identify key genes linked to NAFLD, we initially screened pQTL-associated plasma proteins and validated them using the GepLiver database by comparing normal and NAFLD liver samples (Supplementary Table S11). Given the well-established link between NAFLD and hepatocellular carcinoma, we further refined the candidate list through analysis of the GSE164760 dataset, which compared liver tissues from patients with non-alcoholic steatohepatitis (NASH) and NASH-associated hepatocellular carcinoma (NASH-HCC; Supplementary Table S12). Based on these integrated analyses, four candidate genes were identified: ANGPT1, SKAP2, SPARC, and STAMBP. Quantitative real-time PCR (qPCR) analysis showed that the hepatic mRNA expression levels of these genes were significantly elevated in liver tissues from the HFD group compared to controls. Importantly, *Lactobacillus salivarius* supplementation significantly reduced the expression of these genes relative to the HFD group, suggesting that *L. salivarius* may mitigate NAFLD progression by modulating the expression of key pathogenic genes (Figure 6D).

## 4 Discussion

This study elucidates the causal roles of gut microbiota, protein-to-protein ratios, and plasma proteins in NAFLD using MR analysis and murine models. MR identified 19 gut microbes and 148 protein-to-protein ratios with significant associations with NAFLD, highlighting the roles of gut dysbiosis and systemic protein homeostasis in disease progression. Additionally, 38 plasma proteins were linked to NAFLD, with ANGPT1, SKAP2, SPARC, and STAMBP notably upregulated in NAFLD tissues. Importantly, their expression levels were reduced following *Lactobacillus salivarius* intervention, further supporting their pathogenic relevance. Functional validation in HFD-induced murine NAFLD models showed that *L. salivarius* alleviated hepatic lipid accumulation, improved glucose-lipid metabolism, and reduced inflammation. These findings provide novel insights into the gut microbiota-protein metabolism-NAFLD axis and support microbiome-based interventions for NAFLD.

Although our MR analysis identified a nominal association between *Klebsiella pneumoniae* and NAFLD (*p* = 0.018), this association did not remain statistically significant after correction for multiple testing (FDR = 0.91). Therefore, the potential involvement of *Klebsiella pneumoniae* in NAFLD pathogenesis should be interpreted with caution. *Klebsiella pneumoniae* is a highly adaptive, endotoxin-producing bacterium, and previous studies have implicated it in gut barrier dysfunction, systemic endotoxemia, and activation of the TLR4/NF-κB inflammatory cascade, which may contribute to hepatic steatosis, fibrosis, and metabolic dysfunction (28). Its enrichment in NAFLD patients and its ability to exacerbate liver injury through persistent low-grade inflammation highlight its potential as a microbial target for therapeutic intervention (29). However, further studies are needed to validate the association between *Klebsiella pneumoniae* and NAFLD and to assess its viability as a therapeutic target.

Conversely, *Lactobacillus salivarius* exhibited a significant negative correlation with NAFLD, suggesting a protective role in maintaining metabolic homeostasis. Previous studies have reported similar protective effects of *L. salivarius* in NAFLD models using hens, demonstrating improvements in hepatic lipid metabolism and inflammation through modulation of SREBP1 pathways (30). Experimental validation in HFD-induced murine models demonstrated that *L. salivarius* supplementation effectively reduced hepatic lipid accumulation, improved glucose-lipid metabolism, and suppressed inflammatory cytokine expression. Mechanistically, *Lactobacillus salivarius* is known to modulate gut microbiota composition, enhance intestinal barrier integrity, and regulate bile acid metabolism, thereby attenuating hepatic lipogenesis, insulin resistance, and inflammatory signaling (31). These findings position *Lactobacillus salivarius* as a promising microbiota-based intervention for NAFLD, reinforcing the potential of gut microbiome modulation as a therapeutic avenue for metabolic liver diseases.

Our study identified ANGPT1, SKAP2, SPARC, and STAMBP as causally linked to NAFLD through MR analysis. The qPCR validation in HFD-induced murine models confirmed their upregulation in NAFLD liver tissues, whereas *Lactobacillus salivarius* supplementation markedly reduced their hepatic expression levels. ANGPT1, a key regulator of vascular homeostasis, may promote NAFLD progression by modulating hepatic angiogenesis and endothelial dysfunction, exacerbating steatosis and fibrosis (32). Src kinase-associated phosphoprotein 2 (SKAP2), a cytosolic adaptor protein enriched in myeloid cells, enhances macrophage activation and infiltration via integrin signaling, thereby promoting hepatic inflammation in NAFLD (33). SPARC, a matricellular protein, is upregulated in response to hepatic injury and plays a key role in extracellular matrix remodeling, fibrogenesis, and inflammasome activation. By promoting IL-1β secretion in macrophages and hepatocytes, SPARC contributes to hepatic inflammation and NAFLD progression (34). Mechanistically, STAMBP stabilizes NALP7 by preventing its lysosomal degradation, enhancing inflammasome activation and IL-1β release in response to TLR signaling (35). This pro-inflammatory role suggests its involvement in hepatic inflammation and fibrosis in NAFLD, making it a potential therapeutic target. Collectively, these genes contribute to NAFLD progression via vascular dysfunction, immune activation, and inflammasome signaling, highlighting their potential as therapeutic targets.

While ANGPT1, SKAP2, SPARC, and STAMBP were identified through MR as upstream drivers of NAFLD, the genes assessed in the *Lactobacillus salivarius* treatment group (SREBP1, ChREBP, ACC, and FAS) are well-characterized downstream regulators of hepatic lipid metabolism. These two gene sets represent distinct yet mechanistically interconnected biological layers. ANGPT1, as a vascular remodeling factor, may influence hepatic lipid metabolism by activating the PI3K/AKT signaling pathway, which has been shown to upregulate lipogenic enzymes such as SREBP1, ACC, and FAS (36, 37). SKAP2 enhances macrophage activation and infiltration, which may promote hepatic lipogenesis indirectly via pro-inflammatory cytokines such as IL-1β that activate SREBP1 and ChREBP in hepatocytes (38–40). SPARC enhances IL-1β secretion through inflammasome activation, which may further contribute to hepatic lipid accumulation during NAFLD progression (34). STAMBP promotes IL-1β release via TLR-dependent inflammasome activation, which may indirectly enhance hepatic lipogenesis through inflammatory signaling involving SREBP1 and FAS (35, 41). Although not all of these MR-identified genes have been mechanistically validated, their upstream effects converge on shared metabolic pathways. This integrated framework offers mechanistic insight into how immune and vascular disturbances may indirectly drive hepatic lipid dysregulation, consistent with the transcriptional responses observed after *Lactobacillus salivarius* intervention.

Despite these significant findings, several limitations should be acknowledged. First, although MR provides strong causal inference, potential confounding factors and population heterogeneity may affect the results. Second, while gene expression validation was performed in murine models, direct validation at the protein-to-protein ratio (PPR) level was not conducted. Finally, differences between murine and human physiology may limit the translatability of the findings. In addition, functional validation of other novel gut microbial taxa identified through MR analysis will be pursued to expand our understanding of microbiota-based interventions for NAFLD. Future research will incorporate 16S rRNA or metagenomic sequencing to characterize gut microbiota changes following *Lactobacillus salivarius* intervention, aiming to clarify whether the observed therapeutic effects are mediated by compositional or functional shifts in the microbiome.

## 5 Conclusion

This study establishes a causal link between gut microbiota, protein-to-protein ratios (PPRs), and NAFLD progression, emphasizing the gut-liver axis as a key regulator of disease. Through Mendelian randomization and experimental validation, we identify specific microbial taxa and key proteins that may serve as biomarkers and therapeutic targets. These findings highlight the potential of microbiota-driven protein alterations in guiding biomarker discovery and microbiota-based interventions for NAFLD prevention and treatment.

## Data Availability

The original data generated and analyzed during this study are included within the article and its Supplementary materials. Transcriptomic data used in this study were obtained from the Gene Expression Omnibus (GEO) under accession number GSE164760. In addition, all R scripts used for data preprocessing, statistical analysis, and figure generation have been deposited in a publicly accessible GitHub repository: https://github.com/leileiviviane-lei/GM473_ppr_MR. For further inquiries or requests, please contact the corresponding author.
