# Supplementary material for: Gut microbiota and protein-to-protein ratios in NAFLD: insights from Mendelian randomization and murine studies
Source: Front Nutr. 2025 Jul 18;12:1597390. doi: 10.3389/fnut.2025.1597390 (PMC12313671; doi:10.3389/fnut.2025.1597390)
Supplement: Supplementary file 1 [file Data_Sheet_1.zip › Supplementary_file_1_updated/Supplementary Figure 1.pdf]

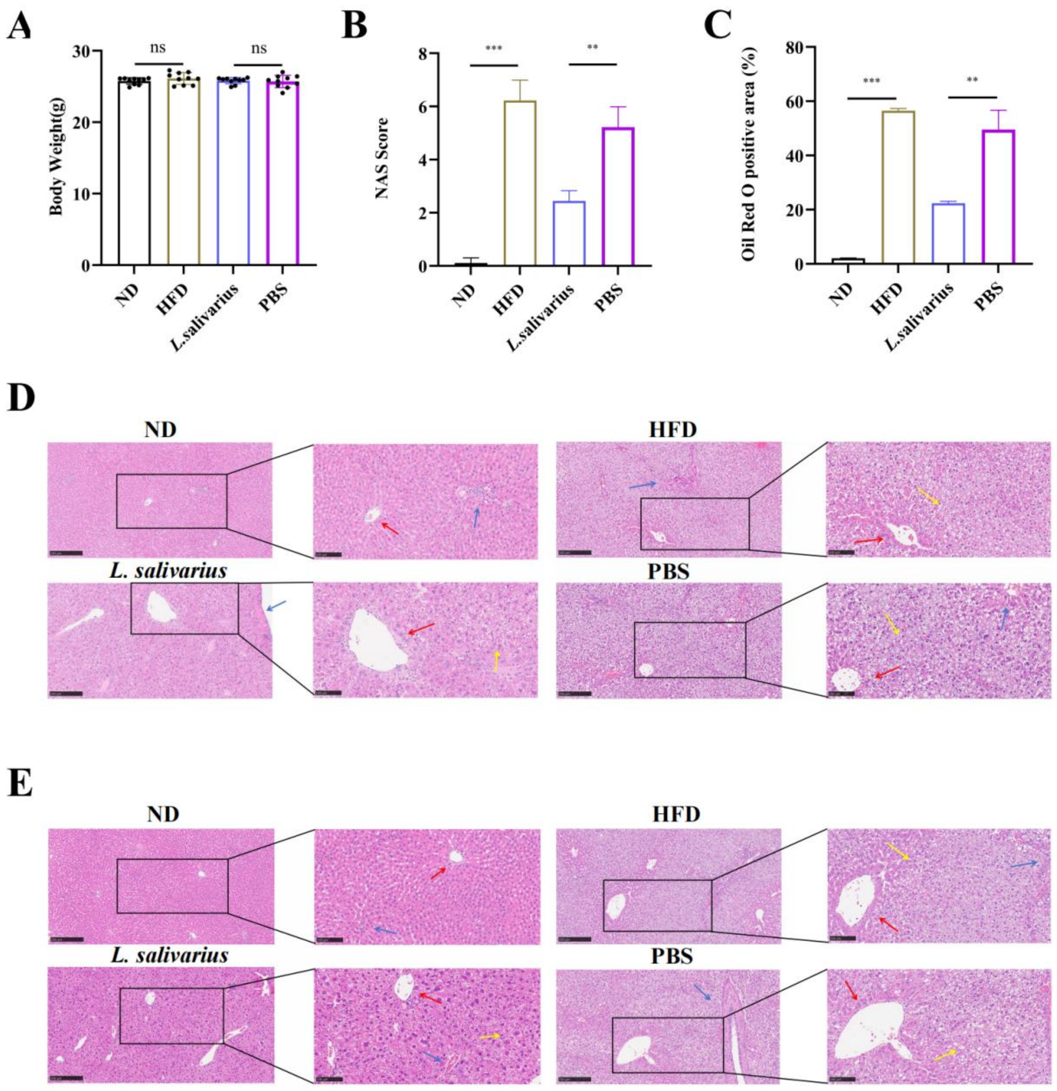

Supplementary Figure 1. Extended histological and lipid accumulation analysis following *Lactobacillus salivarius* treatment. **(A)** Body weight following 7 days of adaptation (N = 10 per group). (**B**) Histological scoring of liver sections according to the NAFLD Activity Score (NAS), based on steatosis, lobular inflammation, and hepatocyte ballooning, assessed from H&E staining. (**C**) Quantification of hepatic lipid accumulation by Oil Red O staining. Data are presented as mean ± SEM (N = 3 per group). Statistical significance: $^{**}p < 0.01$, and $^{***}p < 0.001$, ns: not significant. **(D,E)** Representative hematoxylin and eosin (H&E) staining images of liver sections from two additional mice per group, distinct from the one shown in Figure 6A. Central vein (red arrows), portal triad (blue arrows), and hepatic steatosis (yellow arrows) are indicated. Images were captured at 10× (overview) and 20× (magnification). Scale bars: 250 µm (10×) and 100 µm (20×).
