# Supplementary material for: Gut microbiota and protein-to-protein ratios in NAFLD: insights from Mendelian randomization and murine studies
Source: Front Nutr. 2025 Jul 18;12:1597390. doi: 10.3389/fnut.2025.1597390 (PMC12313671; doi:10.3389/fnut.2025.1597390)
Supplement: Supplementary file 1 [file Data_Sheet_1.zip › Supplementary_file_1_updated/Supplementary Material 2.DOCX]

# Supplementary Material 2

## Experimental validation in mice

### 1 **Preparation of bacterial strains and animal experiments**

*Lactobacillus salivarius* Li01 (CGMCC 7045) was initially isolated from healthy human fecal samples and subsequently stored in the China General Microbiological Culture Collection Center (CGMCC). For this experiment, the bacterial strain was directly acquired from the CGMCC to ensure its purity and authenticity. Upon receipt, the bacteria were anaerobically cultivated in de Man, Rogosa, and Sharpe (MRS) broth to sustain their biological activity and viability.

Forty female SPF-grade C57BL/6 mice were purchased from Beijing Hua Fukang Biotechnology Co., Ltd. All mice were maintained under controlled conditions (temperature, humidity) in an SPF environment and allowed unlimited access to standard food and water. Following an adaptation period of 7 days, mice were randomly assigned into four groups (10 mice per group): ND group (normal diet), HFD group (high-fat diet), *L. salivarius* group (HFD diet plus *Lactobacillus salivarius*), and PBS group (HFD diet plus PBS). The fat content of the HFD was 40%. Mice in the *L. salivarius* and PBS groups were administered bacterial suspension or PBS (10^9 CFU/ml, 0.2 ml per mouse) intragastrically, three times per week for eight consecutive weeks. After continuous high-fat feeding for 20 weeks. All animal experiments were approved by the Ethics Committee of Guangxi Zhuang Autonomous Region People's Hospital (Approval Number: KY-GZR-2024-093). Mice were anesthetized with isoflurane and euthanized with carbon dioxide to minimize pain and ensure a humane end.

### 2 Serological analysis

Commercial kits from Nanjing Jiancheng Bioengineering Institute were used to measure serum ALT, AST, triglycerides (TG), and total cholesterol (TC) concentrations. Liver triglyceride and total cholesterol contents were quantified using commercial assay kits (Nanjing Jiancheng Bioengineering Institute), and the values were normalized to liver protein concentrations measured using a BCA assay kit (Beyotime Biotechnology). Fasting insulin (FINS) levels were measured using an Insulin ELISA kit from Jianglai Bio to calculate the insulin resistance index (HOMA-IR). Inflammatory cytokine levels, including tumor necrosis factor-α (TNF-α), interleukin-6 (IL-6), IL-17A, and IL-10, were detected using ELISA kits from Thermo Fisher Scientific.

### 3 Histological analysis

Liver tissues were collected and fixed in 4% paraformaldehyde, followed by dehydration and paraffin embedding. Tissue sections (5 μm thick) were prepared for histological analysis. Hepatic steatosis, inflammation, and fibrosis were assessed using hematoxylin-eosin (H&E) staining. Steatosis severity was evaluated based on lipid droplet distribution, and fibrotic changes were quantified by measuring fibrotic area. The NAFLD Activity Score (NAS) was calculated by scoring steatosis (0-3), lobular inflammation (0-3), and hepatocyte ballooning (0-2), with a total score ranging from 0 to 8. A NAS of 5 or higher was considered indicative of definite NASH.

### 4 Oil Red O staining and quantification

Oil Red O staining was used to evaluate lipid accumulation in the liver. Frozen tissue sections and hepatocytes were prepared and stained with a modified Oil Red O staining solution (Solarbio, #G1261). The corresponding areas of positive staining were quantified using ImageJ software (version 1.54, National Institutes of Health, USA), and the results were expressed as the percentage of the total tissue area.

### 5 Western blot analysis

Total protein was extracted using lysis buffer supplemented with phosphatase inhibitors. Protein samples were separated via SDS-polyacrylamide gel electrophoresis (SDS-PAGE) and subsequently transferred onto polyvinylidene fluoride (PVDF) membranes. After blocking with skim milk, membranes were incubated overnight at 4°C with specific primary antibodies directed against target proteins (SREBP1, ChREBP, ACC, FAS, and GAPDH, Abcam, UK). Following incubation with secondary antibodies, protein signals were detected using enhanced chemiluminescence (ECL), and densitometric analysis was conducted with ImageJ software.

### 6 RNA extraction and qRT-PCR

Total RNA was extracted from tissues following the manufacturer’s guidelines using commercial extraction kits. Subsequently, RNA samples were converted into complementary DNA (cDNA) utilizing the High Capacity cDNA Reverse Transcription Kit (Invitrogen). Gene expression was quantified through quantitative real-time PCR (qRT-PCR) using SYBR Green PCR Master Mix (Applied Biosystems) and analyzed with a StepOne Real-Time PCR System (Applied Biosystems).

**Table Real-time PCR primers**

| Genes (Species) | Forward (5’-3’) | Reverse (5’-3’) |
| --- | --- | --- |
| SPARC (Mouse) | TGGGAGAATTTGAGGACGGTG | GAGTCGAAGGTCTTGTTGTCAT |
| STAMBP (Mouse) | CCAAGACCGGGTGAGGATTC | CCATGCGGATGATCTCAACAC |
| ANGPT1 (Mouse) | ATCCCGACTTGAAATACAACTGC | CTGGATGATGAATGTCTGACGAG |
| SKAP2 (Mouse) | TAGGAACCTGTTGGCAGATGT | TCTGAGGCTAAGGAAATCGTGT |
